# Transferrin Receptor 1-Associated Iron Accumulation and Oxidative Stress Provides a Way for Grass Carp to Fight against Reovirus Infection

**DOI:** 10.3390/ijms20235857

**Published:** 2019-11-22

**Authors:** Quanyuan Wan, Zhiwei Liao, Youliang Rao, Chunrong Yang, Jianfei Ji, Xiaohui Chen, Jianguo Su

**Affiliations:** 1 College of Fisheries, Huazhong Agricultural University, Wuhan 430070, China; wqyuan_1314@126.com (Q.W.); liaozhiwei1991@163.com (Z.L.); raoyouliang@163.com (Y.R.); jjf1000@163.com (J.J.); 2 Laboratory for Marine Biology and Biotechnology, Pilot National Laboratory for Marine Science and Technology (Qingdao), Qingdao 266237, China; 3 College of Veterinary Medicine, Huazhong Agricultural University, Wuhan 430070, China; chryang@mail.hzau.edu.cn; 4 College of Animal Science and Technology, Northwest A&F University, Yangling, Xianyang 712100, China; cxh889911@163.com

**Keywords:** grass carp (*Ctenopharyngodon idella*), GCRV, iron metabolism, *TfR1*, oxidative stress

## Abstract

Iron is an essential element, closely linked with host immune responses. Nevertheless, the relationship between iron metabolism and virus infection is still unclear in aquatic vertebrates. To address this issue, we employed grass carp (*Ctenopharyngodon idella*) and its lethal virus, grass carp reovirus (GCRV), a double-strand RNA virus, as models. Our results demonstrate that GCRV infection increases the iron content and alters the expression of iron metabolism-related genes both in vivo and in vitro. Of note, the expression of *C. idella* *transferrin receptor 1* (*CiTfR1*) rather than *transferrin* is upregulated upon GCRV infection. To clarify the implications of *CiTfR1* upregulation for antiviral immunity, we proved that *CiTfR1* was not a helper for GCRV invasion, but instead, it inhibited GCRV infection and promoted cell proliferation by facilitating the accumulation of intracellular labile iron pool (LIP), which increases intracellular oxidative stress. Interestingly, we found that *CiTfR1* overexpression inhibited the mRNA expression of *C. idella interferon 1* (*CiIFN1*) and *CiIFN3*. The present study reveals a novel antiviral defense mechanism in teleost where *TfR1* induces the accumulation of LIP, leading to the suppression of virus infection and the proliferation of host cells, indicating that iron can be used as a medicated feed additive for the control of animal viral disease.

## 1. Introduction

To defend against the invasion of various pathogens, vertebrates are equipped with well-developed defensive systems: antibacterial or antiviral properties of tissue fluids and the phagocytic abilities of cells, also known as the immune system including innate and adaptive immunity [1]. The innate immune system, which is initiated by the recognition of pathogen-associated molecular patterns (PAMPs) through germline-encoded pattern-recognition receptors (PRRs), is the first line of host defense against invading pathogens [2]. Theses PAMP-activated PRRs can trigger a series of signaling cascades that rapidly induce the expression of a variety of cytokines involved in the inflammatory and immune responses. Consequently, pathogens are suppressed by these cytokines or directly phagocytized by cytokine-activated macrophages/monocytes. On the other hand, since the prerequisite of infectious diseases is the successful proliferation of pathogens in host cells or tissues, host nutrients used by parasitic pathogens are vital to the disease outbreak. As such, nutrient limitation is another important immune response against the invasion of pathogens, which is termed nutritional immunity [3,4,5].

Since iron (Fe) is a trace element which can switch between two thermodynamically stable oxidation states, namely, ferric iron (Fe^3+^) and ferrous iron (Fe^2+^), it can be incorporated into some proteins whose activities require the transfer of electrons to serve as an ideal redox catalyst [4]. Divalent iron is intimately involved in numerous vital biological processes ranging from energy generation, DNA biosynthesis, and replication to oxygen transport and protection against oxidative stress [6]. Therefore, iron is considered to be of crucial importance to the growth of nearly all organisms, including various pathogenic microorganisms. For propagation, pathogens have evolved various metabolic adaptations to plunder available iron from their hosts. For instance, bacteria can use siderophores to capture iron from their hosts [7], while DNA viruses use their proteins to target certain host iron metabolism-related proteins to increase cellular iron content in the hosts [6,8]. To limit microbial iron supply, hosts develop a primitive and straightforward strategy, namely, an iron-withholding strategy, which widely exists in echinoderms [9], mollusks [10], arthropods [11], and vertebrates [12]. Therefore, the success or failure of iron scramble in those infected hosts impacts the pathological outcome.

In mammalian cells, a well-characterized mechanism that refers to the combination between the plasma protein transferrin (*Tf*) and its receptor named Tf receptor 1 (*TfR1*) is responsible for iron uptake [4,13]. *Tf* is a monomeric protein of 76–81 kDa, consisting of two structurally similar lobes (termed N- and C-lobes), each containing a single iron-binding site [14]. In normal plasma (pH = 7.4), Tf can tightly bind two atoms of Fe^3+^. *TfR1* is the receptor of *Tf*, which is a type II transmembrane glycoprotein consisted of a disulfide-bonded homodimer on the surface of various cell types [15]. Each monomer (molecular weight 90–95 kDa) contains a sizeable C-terminal ectodomain involved in the Tf-binding, a single-pass transmembrane domain, and a short intracellular N-terminal domain. Diferric *Tf* can bind *TfR1* readily, and then initiates the clathrin-mediated endocytosis with the assistance of the TfR trafficking protein [16]. With the entrance of protons, the pH in endosome containing diferric Tf/TfR1 complex decreases, resulting in a conformational change in Tf and release of Fe^3+^ [17]. Subsequently, the apo-Tf/TfR complex returns to the cell surface for the next cycle, whilst Fe^3+^ is reduced to Fe^2+^ by a reductase named six-transmembrane epithelial antigen of the prostate 3 (*STEAP 3*) and then transported into cytoplasm by the ferrous iron transporter divalent metal-ion transporter 1 (*DMT1*, also known as natural resistance-associated macrophage protein 2) [13]. Although intracellular iron serves as the cofactors of various enzymes involved in DNA synthesis, replication, repair, and transcription, it can result in cellular toxicity and ferroptosis as well [18,19]. Fe^2+^ participates in Fenton reaction to generate reactive oxygen species (ROS) to damage lipids, DNA, and proteins [20]. Therefore, the excess iron is frequently stored in ferritin, a primary iron storage protein involved in the iron-withholding strategy [12]. In this process of iron regulation, some cytokines, such as IL-6, TNF-α, and IFN-γ, were reported to regulate the expression of iron metabolism-related genes (IMRGs) [4].

Iron metabolism in teleost and the relationship between iron metabolism and immunity to infections in teleost have been investigated. However, nearly all the studies have focused on the functions of IMRGs in bacterial infection. For instance, Tf [21,22], ferritin [23,24], and hepcidin [25,26] were previously identified to play positive roles against bacterial infection in teleost, whereas the iron-withholding strategy in teleost against virus infection was rarely reported. In view of the influence of *Piscine orthoreovirus* (PRV) on the host’s iron metabolism [27], it is of great importance to clarify the relationship between aquatic virus infection and the iron metabolism, which may contribute to illuminating the antiviral iron-withholding strategies in aquatic animals and exploiting iron-related drugs or feed additives for the prevention and control of viral diseases. By using transcriptome sequencing technology, a previous study reported that the infection of grass carp reovirus (GCRV) affected the iron homeostasis in grass carp (*Ctenopharygodon idella*), a crucial economic fish in China, at the early stage of infection [28]. The present study focused on the relationship between GCRV infection and iron metabolism in *C. idella*. The effect of GCRV infection on the iron contents and the expression of major IMRGs in vivo and in vitro was tested. Since GCRV is a kind of double-strand RNA (dsRNA) virus which does not need Fe to activate deoxyribonucleotides for their replication, we hypothesize that the iron-withholding strategy is inapplicable for grass carp to fight against GCRV infection. The further exploration confirmed the hypothesis that *C. idella TfR1* (*CiTfR1*)-associated iron-accumulation is an effective part in the antiviral response in grass carp.

## 2. Results

### 2.1. GCRV Infection Affects Iron Metabolism In Vivo and In Vitro

**Figure 1:**
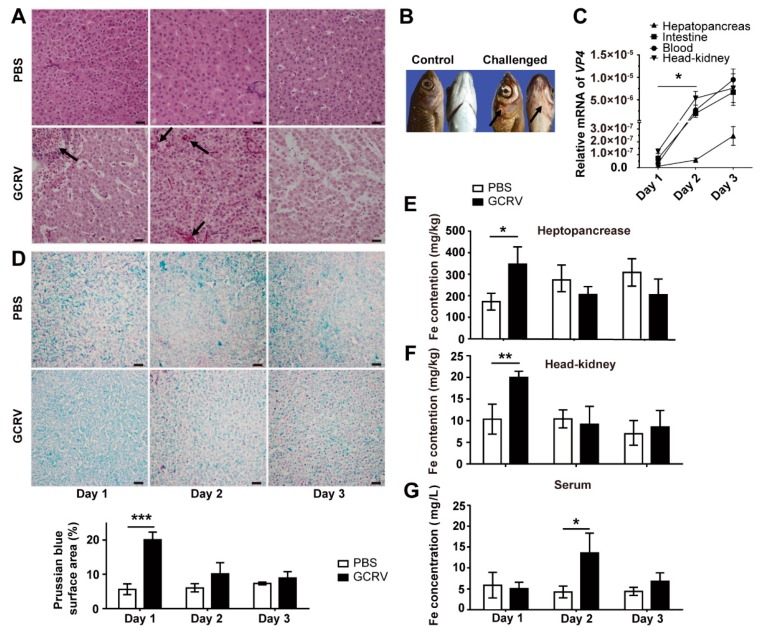
The iron contents in hepatopancreas, head/kidney, and serum in grass carp after grass carp reovirus (GCRV) infection. (**A**) Hepatopancreas damage in infected fish was detected using hematoxylin–eosin (HE) staining. Samples were collected and fixed at the indicated time points postchallenge. Arrows show the hepatic sinus hyperemia and the hydropic degeneration of hepatocytes. Bar = 20 μm. (**B**) The symptoms of the GCRV-challenge test in grass carp. Arrows show hemorrhage sites at branchiostegite of infected fish. (**C**) mRNA expression levels of the *VP4* gene of GCRV in hepatopancreas, intestine, blood, and head/kidney of infected fish were monitored. Data are presented in relative expression units where *18S rRNA* was used to normalize all samples. (**D**) Iron in hepatopancreas was stained by Prussian blue, and nuclei were stained with fast red. Samples were collected and fixed at the indicated time points postchallenge. Bar = 20 μm. Color density values were quantified by using ImageJ software. (**E**,**F**) The iron content in hepatopancreas (**E**) and head/kidney (**F**), detected by using ICP-OES. Samples were collected at the indicated time points postchallenge and then digested by using microwave for the iron content assay. (**G**) The iron content in serum, detected by using an automatic biochemistry analyzer. A total of 180 fish were used for each independent experiment. Data represent mean ± SD of three independent experiments. * *p* < 0.05, ** *p* < 0.01, *** *p* < 0.001.

To clarify the relationship between iron and the antiviral response in grass carp, we challenged grass carp with GCRV, as detailed in the Materials and Methods section. Before the challenge experiment, we verified that the serum iron content of fish in the experimental group was the same as that in the control group. By observing the typical hemorrhagic symptoms, the increased mRNA level of viral *VP4* gene in fish, and pathological alterations in the hepatopancreas tissues, we confirmed that experimental fish were successfully infected by GCRV, as seen in Figure 1A–C. On the contrary, no hemorrhagic symptom was observed, and the mRNA of *VP4* gene could not be detected in control fish. Subsequently, iron contents in those collected samples, including hepatopancreas (a specific tissue mixed with formless liver and en masse pancreas in *Cyprinid* fish), blood, and head/kidney, were measured. The subsequent Prussian blue staining assay and coupled plasma optical emission spectrometry (ICP-OES) results revealed that iron content in the hepatopancreas and head/kidney of challenged fish was significantly increased at day 1 postinfection (p.i.), compared to that of the unchallenged fish (*p* < 0.05), as seen in Figure 1D–F. We also found that the serum iron content in challenged fish increased at 2 d p.i., compared to that in the control group (*p* < 0.05), as seen in Figure 1G.

**Figure 2:**
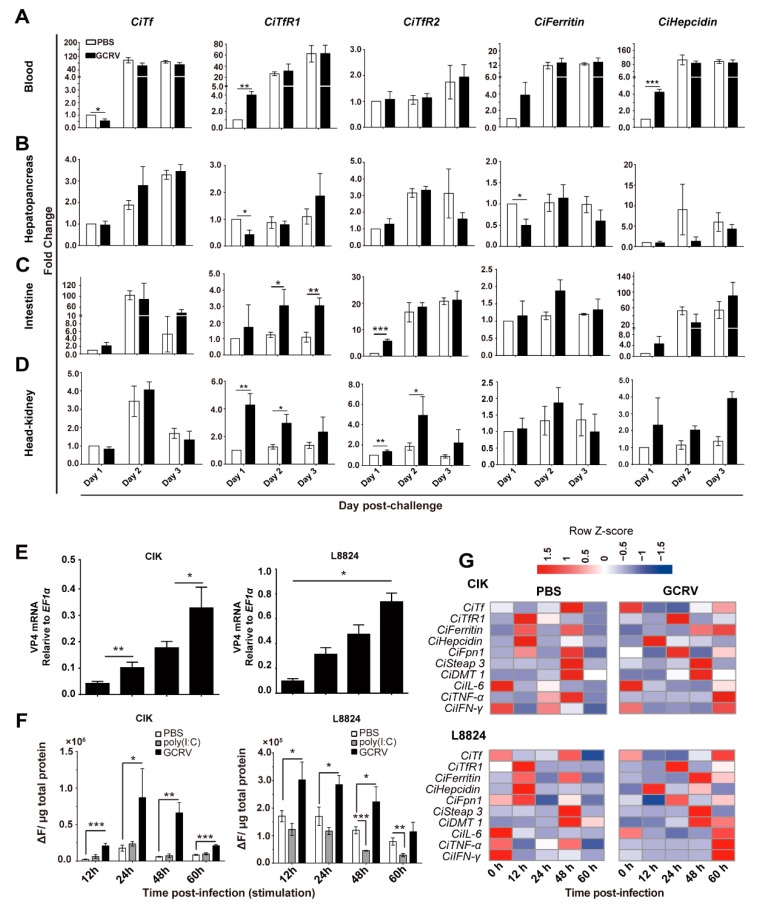
GCRV infection affects intracellular iron accumulation and the expression of iron metabolism-related genes (IMRGs). (**A**–**D**) GCRV infection influences the expression of IMRGs in vivo. Blood, hepatopancreas, intestine, and head/kidney samples were gathered at the indicated time points after GCRV injection. The relative mRNA expression levels of *CiTf*, *CiTfR1*, *CiTfR2, CiFerritin*, and *CiHepcidin* in blood (**A**), hepatopancreas (**B**), intestine (**C**), and head/kidney (**D**) were detected, respectively. Y-axis represents the relative fold change. (**E**) The relative mRNA levels of *VP4* in CIK and L8824 cells after GCRV infection. (**F**) The iron contents in CIK and L8824 cells increase after GCRV infection. Cells were evenly seeded into 24-well plates (5 × 10^5^/well), and then stimulated with poly(I:C) or infected with GCRV (MOI = 1) at 24 h cultivation. At the indicated time points, cells were harvested. The fluorescence value of each well was measured. (**G**) The relative expression profiles of IMRGs and IMRG regulation cytokines in PBS- and GCRV-challenged CIK or L8824 cells. Red indicates the relative high expression, while blue indicates low expression. Heatmap was plotted by using the heatmap.2 package of R program (http://www.R-project.org/). Data are presented in relative expression units where *18S rRNA* (**A**–**D**) or *EF1α* (**E**,**G**) was used to normalize samples. A total of 180 fish were used. Data represent mean ± SD of three independent experiments. * *p* < 0.05, ** *p* < 0.01, *** *p* < 0.001.

Given the effect of GCRV infection on the iron content, the relative mRNA expression levels of representative IMRGs (*CiTf*, *CiTfR1*, *CiTfR2*, *CiFerritin*, and *CiHepcidin*) upon GCRV infection were measured. The results showed that GCRV infection significantly affected the expression of these genes in hepatopancreas, head/kidney, blood, and intestine of *C. idella*, as seen in Figure 2A–D. In detail, the expression of *CiTf* was downregulated in blood at 1 d p.i. but upregulated in the intestine at 3 d p.i. (*p* < 0.05); that of *CiTfR1* was upregulated in blood and head/kidney, but downregulated in hepatopancreas at 1 d p.i. (*p* < 0.05); that of *CiTfR2* was upregulated in the intestine at 1 d p.i. and in head/kidney at 2 d p.i., but downregulated in hepatopancreas at 3 d p.i. (*p* < 0.05); that of *CiFerritin* was upregulated in blood but downregulated in hepatopancreas at 1 d p.i. (*p* < 0.05); and that of *CiHepcidin* was upregulated in blood at 1 d p.i. but upregulated in hepatopancreas at 2 d p.i. (*p* < 0.05).

Accordingly, the effect of GCRV on iron metabolism was investigated in *C. idella* kidney cell line (CIK) and *C. idella* liver cell lines (L8824) as well. Cells of the experimental group were successfully infected, presenting an increase of the viral *VP4* mRNA, as seen in Figure 2E, while *VP4* mRNA was not detected in the control group. Compared to the control group, the intracellular labile iron pool (LIP) contents displayed different levels of increase upon GCRV infection. Significantly, LIP contents in CIK cells were increased for 8.9-, 5.1-, 10.6-, and 5.6-fold at 12, 24, 48, and 60 h p.i., respectively (*p* < 0.05), as seen in Figure 2F, and that in L8824 cells was increased for 1.78-fold at 12 h p.i. (*p* < 0.05), as seen in Figure 2F. However, it was not affected by the stimulation of polyinosinic:polycytidylic acid [poly(I:C)] (a dsRNA simulant for provoking innate immune signaling pathways) in CIK cells, and even decreased in L8824 cells for 2.8-fold and 2.6-fold at 48 and 60 h p.i., respectively. Furthermore, we found that GCRV infection altered the expression patterns of IMRGs in CIK cells as well, as seen in Figure 2G.

Since the macrophage is the primary cell that regulates the iron homeostasis [29], we investigated the effect of GCRV infection on the iron metabolism of *C. idella* macrophages in vitro. Macrophages of grass carp was isolated from the head/kidney, as seen in Figure S1A. Subsequent results showed that GCRV infected macrophage, as seen in Figure S1B, and that the LIP content in macrophage was significantly increased at 12 h p.i. (*p* < 0.05), as seen in Figure S1C. Moreover, the expression of IMRGs was affected by GCRV infection at 12 h p.i. in macrophages as well, as seen in Figure S1D. Specifically, the expression of *CiTf* in the macrophages was not affected, but those of *CiTfR1*, *CiTfR2*, and *CiHepcidin* were significantly upregulated (*p* < 0.05), while those of *CiFerritin* and *CiFerroportin1* (*CiFpn1*) were significantly downregulated (*p* < 0.05). Taken together, this observation illustrates that GCRV infection can affect iron metabolism in *C. idella* and disrupt the expression patterns of IMRGs in vivo and in vitro.

### 2.2. GCRV Infection Induces the Expression of CiTfR1

**Figure 3:**
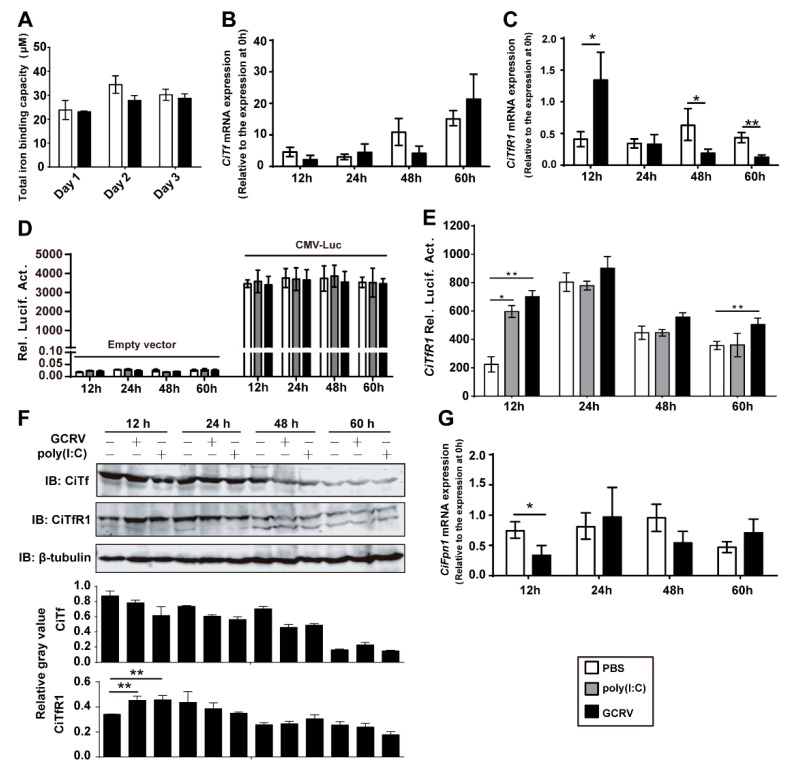
The expression of *CiTfR1* rather than *CiTf* is enhanced at the early stage of GCRV infection. (**A**) The total iron-binding capacity (TIBC) in grass carp serum was detected by using an automatic biochemistry analyzer. (**B**,**C**,**G**) The relative mRNA expression levels of *CiTf* (**B**), *CiTfR1* (**C**), and *CiFpn1* (**G**) in CIK cells after GCRV infection. CIK cells were seeded into 12-well plates (1× 10^6^/well) 24 h before GCRV infection. The experimental group was infected with GCRV, while the control group was treated with PBS. Then, cells were harvested at the indicated time points. Data are presented in relative expression units where *EF1α* was used to normalize samples. The expression level of each gene in CIK cells at 0 h was deemed as standard level (relative level is 1). (**D**,**E**) The effects of GCRV infection or poly(I:C) stimulation on the promoter activity of the *CiTfR1* gene. In total, CIK cells were seeded into 24-well for 24 h cultivation (5 × 10^5^/well), and then cells were cotransfected with 38 ng pRL-TK and 760 ng pGL3-basic, pCMVpro-Luc (**D**), or pTfR1pro-Luc (**E**). Twenty-four hours later, the cells were infected with GCRV or treated with PBS or poly(I:C), and then cells were harvested at the indicated time points for the detection of luciferase activities. (**F**) The effects of GCRV infection or poly(I:C) stimulation on the protein levels of *CiTf* and *CiTfR1* genes. CIK cells were seeded in six-well plates for 24 h cultivation (3 × 10^6^/well), and then the cells were infected with GCRV or treated with PBS or poly(I:C). At the indicated time points, cells were harvested, and the cell lysates were used for WB analysis. Data represent mean ± SD of three independent experiments. * *p* < 0.05, ** *p* < 0.01.

Considering the pivotal role of *TfR1* in iron metabolism, we questioned whether the increase of iron content in vivo and in vitro is associated with the iron transport initiated by *CiTf* and *CiTfR1*. To assess the protein level of *CiTf* in blood, we measured the serum total iron-binding capacity (TIBC) in blood samples. The results showed that *CiTf* protein in blood was not significantly influenced by GCRV infection (*p >* 0.05), as seen in Figure 3A. Furthermore, to confirm the effect of GCRV infection on *CiTf* and *CiTfR1*, we also measured mRNA expressions in vitro, and found that the expression of *CiTf* was not affected by GCRV infection as well (*p* > 0.05), as seen in Figure 3B, while that of *CiTfR1* was significantly upregulated in CIK cells at 12 h p.i. (*p* < 0.05), as seen in Figure 3C. To further confirm the result, we employed the dual-luciferase reporter system. To identify the promoter activity of the 5′-flanking sequence of *CiTfR1* gene, we transferred the pTfR1pro-EGFP plasmid into CIK cells. The subsequent observation showed that the 5′-flanking sequence of *CiTfR1* gene could promote the expression of the enhanced green fluorescent protein (EGFP), as seen in Figure S2, suggesting that the 5′-flanking sequence can be regarded as a promoter of *CiTfR1* gene. Additionally, the promoter activities of empty vector (negative control), namely pGL3-Basic, and pCMV-Luc (positive control) were identified, which showed that there was almost no luciferase activity in the empty vector-transfected cells, while strong luciferase activity in pCMV-Luc transfected cells, suggesting that the dual luciferase system worked well, as seen in Figure 3D. Based on these identification tests, the results of the dual luciferase reporter assay revealed that GCRV infection could significantly increase the promoter activity of *CiTfR1* gene in CIK cells at 12 and 60 h p.i. (*p* < 0.05), as seen in Figure 3E, further verifying that *CiTfR1* was upregulated during GCRV infection.

To detect the protein levels of *CiTfR1* and *CiTf*, we prepared anti-CiTfR1 and anti-CiTf polyclonal antisera, respectively. The result showed that the *CiTfR1* protein was successfully expressed and purified in vitro, as seen in Figure S3A, and that the anti-CiTfR1 serum rather than the negative (unimmunized) serum could recognize and specifically bind to the cellular protein, showing prominent bands with a molecular mass of between 70 kDa and 100 kDa, as seen in Figure S3B. Since the predicted molecular mass of *CiTfR1* was about 85 kDa, this result implied that the anti-CiTfR1 antiserum was able to detect cellular *CiTfR1*. Furthermore, when we used anti-CiTfR1 antiserum, preincubated with either phosphate buffer solution (PBS) or prokaryotically expressed glutathione sulfhydryl transferase (GST), as primary antibody (Ab) to detect purified recombinant GST-CiTfR1, the target bands were visible, as seen in Figure S3C. However, when we used anti-CiTfR1 antiserum, which was preincubated with prokaryotically expressed GST-CiTfR1, as primary Ab to detect purified recombinant GST-CiTfR1, the target protein bands were non-existent, as seen in Figure S3C. These results proved that the anti-CiTfR1 antiserum was able to recognize *CiTfR1* specifically, and could be employed for further investigation. A similar result was observed when we conducted the same verification test for anti-CiTf antiserum. We also found that *CiTf* was not significantly affected, but *CiTfR1* was significantly increased at 12 h p.i. (*p* < 0.05), upon GCRV infection or poly(I:C) stimulation, as seen in Figure 3F.

Another mechanism relevant to intracellular iron accumulation is iron efflux mediated by the iron exporter, *Fpn1* [30]. Therefore, we also examined the expression change of *CiFpn1* upon GCRV infection in CIK cells and found that the expression of *CiFpn1* was significantly inhibited at 12 p.i., compared to that in control group (*p* < 0.05), as seen in Figure 3G. These results suggested that both *CiTfR1* and *CiFpn1* may be responsible for the iron accumulation upon GCRV infection in CIK cells at the early stage. Taken together, GCRV infection leads to the upregulation of *CiTfR1* and downregulation of *CiFpn1*, but does not significantly affect the expression of *CiTf* at the early stage.

### 2.3. CiTfR1 Promotes Iron Accumulation in CIK Cells upon GCRV Infection

**Figure 4:**
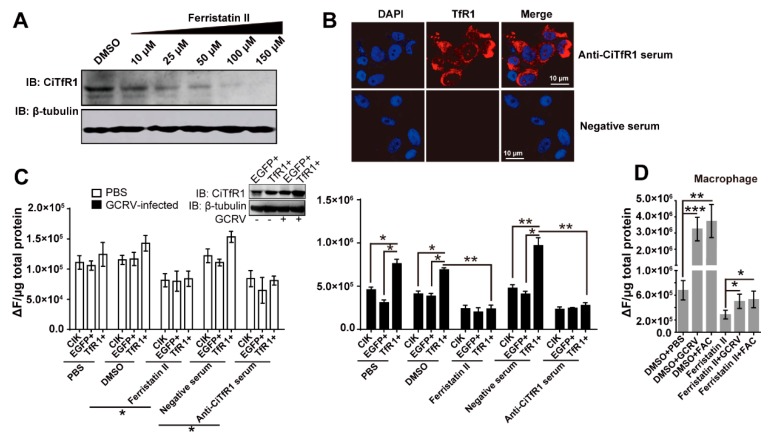
Labile iron pool (LIP) in GCRV-infected CIK cells and macrophages. (**A**) The degradation effect of Ferristatin II on *CiTfR1*. CIK cells were seeded in six-well plates for 24 h cultivation (3 × 10^6^/well), and then the cells were treated with 1 % DMSO or the indicated concentrations of Ferristatin II. Twelve hours later, cells were harvested for WB analysis. (**B**) The verification of *CiTfR1* polyclonal antiserum in CIK cells. Confocal images of *TfR1* in CIK cells at 12 h after incubation with anti-CiTfR1 polyclonal serum or negative serum. Fixed cells were stained for *TfR1* (red) and DAPI (blue). Scale bar = 10 μm. (**C**) Either TfR1+, EGFP+, or CIK cells were evenly seeded into 96-well plates for 24-h cultivation (2 × 10^4^/well), and then cells were treated with either 1 % PBS, 1 % DMSO, 100 μM Ferristatin II, 1% anti-CiTfR1 serum or 1% negative serum. Twelve hours later, all the cultivated cells were infected with GCRV (MOI = 1). Cells were harvested at 12 h postinfection. The intracellular LIP contents were measured by using a Multiscan Spectrum microplate reader. The protein level of *CiTfR1* in EGFP+ and TfR1+ cells were detected by WB analysis. (**D**) Isolated macrophages were seeded into 12-well plates (5 × 10^5^/well), and then treated with the indicated treatments (concentrations of Ferristatin II and sera were 100 μM and 1%, respectively; MOI of GCRV is 1). The LIP content of each well was measured at 12 h post-treatment. Data represent mean ± SD of three independent experiments. * *p* < 0.05, ** *p* < 0.01, *** *p* < 0.001.

Since the expression of *CiTfR1* was increased by GCRV infection, we assumed that *CiTfR1* directly transported iron into cells during GCRV infection. In testing this assumption, Ferristatin II, a degradation-accelerant of *TfR1* was used [31], and its degradation effect on *CiTfR1* was verified at concentrations above 10 μM, as seen in Figure 4A. Moreover, the microscopy images showed that anti-CiTfR1 serum, but not negative serum, could detect the *CiTfR1* protein in the cytoplasm and on the cell membranes, suggesting that the polyclonal anti-CiTfR1 serum could be used for the blockade of *CiTfR1*, as seen in Figure 4B. Besides, we established a *CiTfR1* stable-overexpressed cell line (TfR1+) to investigated the effect of *CiTfR1* overexpression on the LIP content in CIK cells. At the normal condition, the LIP content was not affected by *CiTfR1* overexpression but affected by *CiTfR1* blockade or degradation, as seen in Figure 4C. Upon GCRV infection, the intracellular LIP content in TfR1+ cells was significantly higher than that in EGFP+ cells (*p* < 0.05), as seen in Figure 4C. Meanwhile, *CiTfR1* blockade or degradation impaired the rising of LIP content caused by CiTfR1 overexpression (*p* < 0.05), as seen in Figure 4C. Likewise, we found that *CiTfR1* degradation decreased the LIP content and impaired the rising effect on the LIP content caused by GCRV infection and ferric ammonium citrate (FAC) (a kind of bioavailable iron for the iron supplement) addition in macrophages upon GCRV infection, as seen in Figure 4D. Taken together, these results confirm that *CiTfR1* promotes the iron accumulation in CIK cells upon GCRV infection.

### 2.4. CiTfR1 Is Not a Helper of GCRV Infection

**Figure 5:**
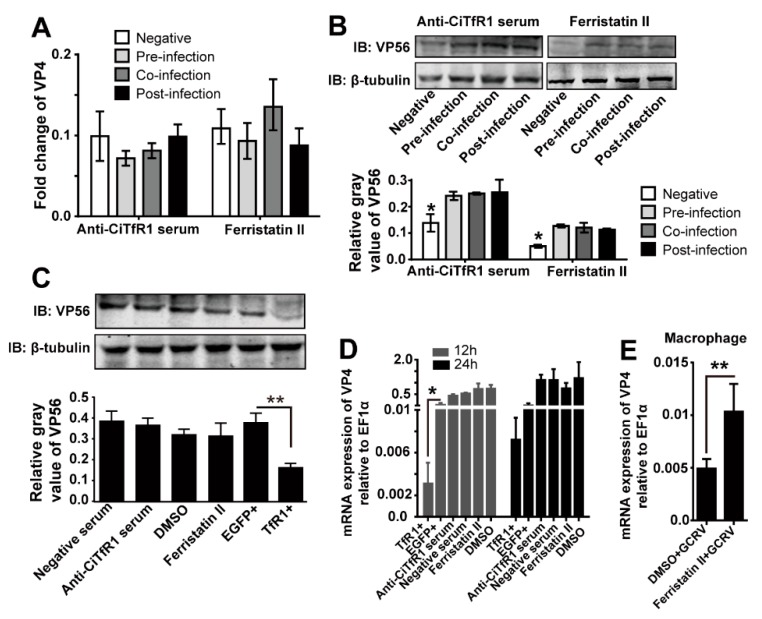
*CiTfR1* is not a helper for GCRV infection. (**A**,**B**) The relative mRNA expression levels of *VP4* (**A**) and the protein levels of *VP56* (B) in PBS, anti-CiTfR1 serum, or Ferristatin II treated and GCRV infected CIK cells. CIK cells cultured in six-well plates were treated with either PBS, 100 μM Ferristatin II, or 1% anti-CiTfR1 serum at 2 h preinfection, coinfection, or 2 h postinfection with GCRV at MOI of 1. Cells were harvested at 12 h p.i. for RNA isolation or WB analysis. (**C**,**D**) The protein levels of *VP56* in the indicated cells were examined at 12 h p.i. The relative gray value of each band in three independent experiments was analyzed (**C**). mRNA expression levels of *VP4* in the indicated cells were examined at 12 and 24 h p.i. (**D**). (**E**) Isolated macrophages were seeded into 12-well plates (5 × 10^5^/well) and then infected with GCRV (MOI = 1) and treated with 1 % DMSO or 100 μM Ferristatin II. mRNA expression levels of *VP4* in the indicated cells were examined at 12 h p.i. Data are presented in relative expression units where *EF1α* was used to normalize all samples. Data represent mean ± SD of three independent experiments. * *p* < 0.05, ** *p* < 0.01.

In consideration of the role of *TfR1* in helping the cell entry of several viruses, including New World hemorrhagic fever arenavirus [32], hepatitis C virus (HCV) [33], and poliovirus [34], we questioned whether *CiTfR1* helps the GCRV infection, thus GCRV coerces the expression of *CiTfR1*, which then promotes the intracellular iron accumulation. To illuminate this, we treated CIK cells with polyclonal anti-CiTfR1 serum or Ferristatin II with three sets, as designed before [33]: treatment prior to GCRV infection (preinfection), treatment together with infection (coinfection), or treatment after infection (postinfection), and compared the GCRV loads among the three sets in CIK cells. The results showed that the GCRV loads in the three sets of treated cells were higher than those in nontreated cells. Importantly, the GCRV load in the pre-infection set was not less than those in coinfection sets or postinfection sets at 12 h p.i. (*p >* 0.05), as seen in Figure 5A,B, indicating that *CiTfR1* degradation before GCRV infection did not inhibit the GCRV infection. Besides, we investigated the effect of *CiTfR1* overexpression or degradation on the GCRV load in CIK cells and macrophages. The results showed that the *CiTfR1* overexpression significantly reduced the protein level of *VP56*, but neither Ferristatin II nor anti-CiTfR1 serum treatment affected the protein level of *VP56* in CIK cells at 12 h p.i. (*p* < 0.05), as seen in Figure 5C. Besides, upon GCRV infection, the mRNA levels of *VP4* in different treated-CIK cells were detected. The results showed that the mRNA level of *VP4* was lower in the TfR1+ cells than in the EGFP+ cells at 12 h p.i. (*p* < 0.05), but the mRNA expression levels were not affected by *CiTfR1* degradation or blockade (*p >* 0.05), as seen in Figure 5D. Additionally, we found that the mRNA levels of *VP4* was significantly increased by *CiTfR1* degradation in macrophages upon GCRV infection as well (*p* < 0.05), as seen in Figure 5E. Taken together, these results reveal that *CiTfR1* does not play an assistant role in GCRV infection.

### 2.5. CiTfR1 Plays a Positive Role in Antiviral Response in CIK Cells

**Figure 6:**
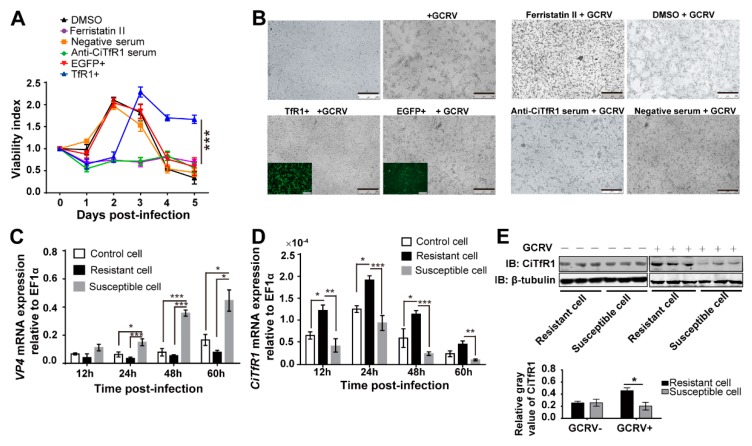
*CiTfR1* plays a positive role in CIK cells against GCRV infection. (**A**,**B**) *CiTfR1* increases the viability of CIK cells against GCRV infection. 1 % DMSO, 100 μM Ferristatin II, 1% negative serum, or 1% anti-CiTfR1 serum treated CIK cells, EGFP+ cells, and TfR1+ cells were infected with GCRV (MOI = 1.5). At the indicated time points, the OD of each well was detected (**A**); At 24 h p.i., cells were imaged by using a fluorescence microscope, bar = 250 μm (**B**). (**C**,**D**) Relative mRNA expression levels of *VP4* (**C**) and *CiTfR1* (**D**) in GCRV-infected control, GCRV-resistant, and GCRV-susceptible CIK cells. Three sorts of CIK cells (1 × 10^6^/sort) were equally seeded into 12-well plates. After 24 h cultivation, cells were infected with GCRV and then harvested at the indicated time points. (**E**) The protein levels of *CiTfR1* in GCRV-susceptible and GCRV-resistant CIK cells treated with GCRV or PBS. The two sorts of CIK cells cultured in six-well plates were treated with GCRV or PBS and harvested for WB analysis at 12 h after the treatment. Data represent the mean ± SD of three independent experiments. * *p* < 0.05, ** *p* < 0.01, *** *p* < 0.001.

Given that the *CiTfR1* did not facilitate the infection of GCRV and that *CiTfR1* suppression increased the intracellular GCRV load, we hypothesized that *CiTfR1* upregulation upon GCRV infection might be a host active defense response, and that those cells highly expressing *CiTfR1* might be more resistant to GCRV infection. Therefore, we sought to validate the positive role of *CiTfR1* in CIK cells against GCRV infection. Upon GCRV infection, the viability index of TfR1+ cells was significantly higher than those of other groups of CIK cells after 3 d p.i. (*p* < 0.05), as well as both anti-CiTfR1 serum and Ferristatin II treatments significantly restrained the viability of CIK cells (*p >* 0.05), as seen in Figure 6A. Likewise, at 24 h post-GCRV infection, by using microphotography, we found that the viability of TfR1+ cells was stronger than that of EGFP+ cells, and that the viability of Ferristatin II- or anti-CiTfR1 serum-treated CIK cells were weaker than that of dimethyl sulfoxide (DMSO)- or negative serum-treated CIK cells, respectively, as seen in Figure 6B. These results demonstrated that *CiTfR1* could protect CIK cells from GCRV infection.

Previously, we sorted CIK cells into two phenotypes against GCRV infection by using flow cytometry, namely GCRV-resistant and -susceptible CIK cells [35]. In the present study, we reconfirmed that the virus loads in GCRV-susceptible CIK cells were higher than those in control and GCRV-resistant CIK cells upon GCRV infection (*p* < 0.05), as seen in Figure 6C. Although the mechanism of phenotypic differential of these cells was found to involve epigenetics and some pathways, including antioxidant activity, we found that the expression level of *CiTfR1* in GCRV-resistant cells was significantly higher than that in GCRV-susceptible cells at 12 h p.i. (*p* < 0.05), as seen in Figure 6D,E, evidencing our assumption that high expression of *CiTfR1* is associated with the resistance of CIK cells against GCRV infection. Taken together, the above findings indicate that *CiTfR1* plays a positive role in the antiviral response against GCRV infection in CIK cells.

### 2.6. CiTfR1 Inhibits the mRNA Level of CiIFN1 and CiIFN3 but Enhances the Intracellular Oxidative Stress

**Figure 7:**
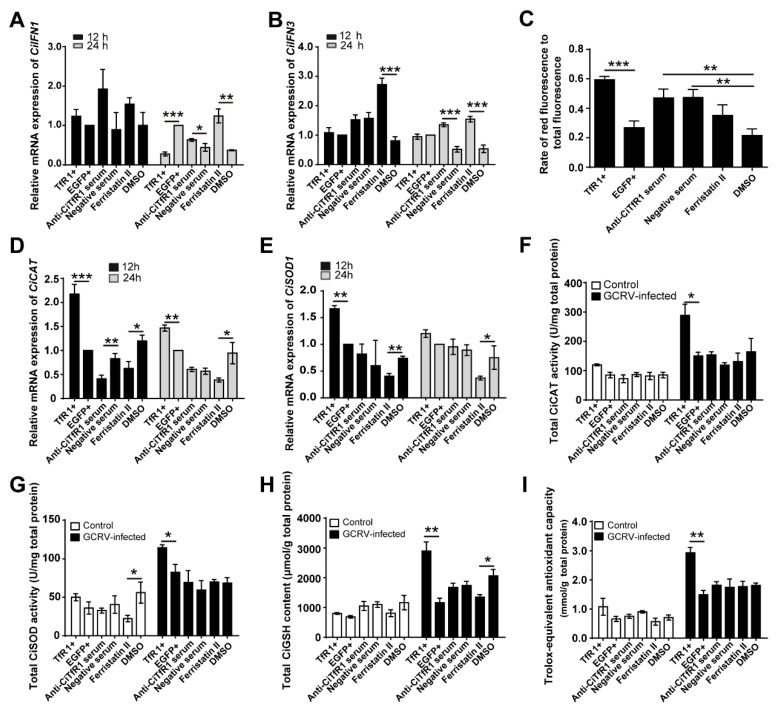
*CiTfR1* inhibits the expression of *CiIFN1* and *CiIFN3* but enhances the intracellular oxidative stress upon GCRV infection. (**A**,**B**,**D**–**I**) Either TfR1+, EGFP+, or CIK cells were equably seeded into 12-well plates for 24 h cultivation (1 × 10^6^/well), and then CIK cells were treated with DMSO, Ferristatin II, 1% anti-CiTfR1 serum or negative serum, respectively. Twelve hours later, all the cultivated cells were infected with GCRV (MOI = 1). Cells were harvested at 12 h and 24 h p.i. for detection of relative mRNA expression of *CiIFN1* (**A**), *CiIFN3* (**B**), *CiCAT* (**D**), and *CiSOD1* (**E**). Data are presented in relative expression units where *EF1α* was used to normalize all samples. Cells were harvested for the detection of total cellular *CAT* (**F**) and *SOD* activities (**G**), Trolox-equivalent antioxidant capacity (**H**), and total *GSH* content (**I**) at 12 h p.i. (**C**) Superoxide anion in differently treated cells was assessed by using DHE method at 12 h p.i. DHE that emits blue fluorescence indicates the total intracellular DHE; ethidium that emits red fluorescence indicates the intracellular superoxide anion. The rate of red fluorescence to the total fluorescence represents the relative amount of intracellular superoxide anion. Data represent mean ± SD of three independent experiments. * *p* < 0.05, ** *p* < 0.01, *** *p* < 0.001.

Based on the above findings, we questioned whether the positive role of *CiTfR1* in the anti-GCRV infection response was associated with the induction of the type I IFN (IFN-I) system. We found that *CiTfR1* overexpression did not increase the mRNA expression of both *CiIFN1* and *CiIFN3*, as seen in Figure 7A,B. Interestingly, *CiTfR1* overexpression inhibited the mRNA expression level of *CiIFN1* significantly at 24 h p.i. (*p* < 0.05), as seen in Figure 7A. Beyond that, mRNA expression levels of *CiIFN1* or/and *CiIFN3* were upregulated by Ferristatin II or anti-CiTfR1 serum treatment in different degrees upon GCRV infection, as seen in Figure 7A,B

We further explored the possible mechanism by which *CiTfR1* improved the viability of CIK cells against GCRV infection. One probable mechanism is that *CiTfR1* increases the iron content in the cytoplasm to enhance oxidative stress through Fenton reaction. To test this hypothesis, we examined the oxidative stress in CIK cells after GCRV infection. We found that the *CiTfR1* overexpression enhanced the intracellular superoxide anion upon GCRV infection (*p* < 0.05), as seen in Figure 7C. Since intracellular superoxide anions are regulated by other molecules, we did not observe the downregulation of intracellular superoxide anion after *CiTfR1* blockade or degradation. In contrast, we found that anti-TfR1 serum and negative sera enhanced the intracellular superoxide anion. Since these sera were harvested from immunized rabbits, they should contain various cytokines which were responsible for the increased intracellular superoxide anion [36]. Besides, at 12 h p.i., we found that mRNA expression levels of *C. idella catalase* (*CiCAT*) and *C. idella superoxide dismutase 1* (*CiSOD1*) in TfR1+ cells were significantly higher than those in normal CIK cells, and that mRNA expression levels of *CiCAT* and *CiSOD1* in Ferristatin II-treated CIK cells were significantly lower than those in DMSO-treated CIK cells (*p* < 0.05), as seen in Figure 7D,E. At 12 h p.i., the mRNA expression level of *CiCAT* was downregulated by anti-CiTfR1 serum treatment (*p* < 0.05), as seen in Figure 7D. We found that total activities of *CAT* and *SOD*, total glutathione (*GSH*) content, and total antioxidant capacity in TfR1+ cells were significantly higher than those in normal CIK cells, but were not affected by Ferristatin II or anti-CiTfR1 serum treatments upon GCRV infection, as seen in Figure 7F–I. Similar results were found in macrophages, where the mRNA levels of *CiSOD1* and *CiCAT* were upregulated upon GCRV infection or FAC addition, but this effect was impaired by *CiTfR1* degradation, as seen in Figure S4. Collectively, these results illustrate that the positive role of *CiTfR1* in anti-GCRV infection response is related to the intracellular oxidative stress, but not the IFN-I system.

### 2.7. Iron Inhibits the Replication of GCRV and Enhances the Intracellular Oxidative Stress

**Figure 8:**
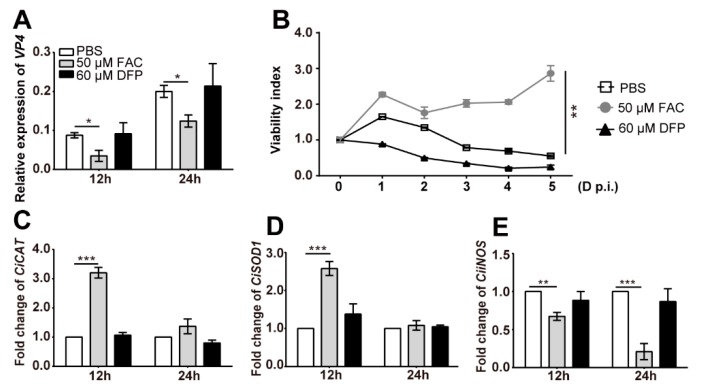
Iron reduces the load of GCRV, enhances oxidative stress in CIK cells, and improves cell survival. CIK cells were seeded into 12-well (1 × 10^6^/well) (**A**,**C**,**D**) or 96-well plates (1 × 10^4^/well) (**B**), respectively, for 24 h cultivation, and then infected with GCRV (MOI = 1). Simultaneously, PBS, FAC (final concentration is 50 μM), and DFP (final concentration is 60 μM) were added into the corresponding wells. At the indicated time points, cells were harvested for RNA isola4040tion, and then the relative expression levels of *VP4* (**A**), *CiCAT* (**C**), *CiSOD1* (**D**), and *CiiNOS* (**E**) were detected. Data are presented in relative expression units where *EF1α* was used to normalize all samples. At the indicated days, the viability index of each well was measured by using the MTT method (**B**). Data represent mean ± SD of three independent experiments. * *p* < 0.05, ** *p* < 0.01, *** *p* < 0.001.

To verify that the intracellular oxidative stress caused by *CiTfR1* is associated with the iron accumulation in the cytoplasm, we further investigated the impact of iron on GCRV replication and intracellular oxidative stress in CIK cells. As expected, GCRV replication was significantly impeded in CIK cells cultured in the medium containing 50 μM of FAC, comparing to that in medium either containing 50 μM of PBS or 60 μM of deferiprone (DFP), a kind of iron chelator for the iron-deprivation, suggesting that iron suppressed the GCRV replication in CIK cells, as seen in Figure 8A. Likewise, we found that 50 μM FAC, rather than 60 μM DFP, was conducive to enhance the viability of CIK cells upon GCRV infection, as seen in Figure 8B.

Then, we investigated whether the iron addition also led to intracellular oxidative stress. After being cultured in mediums containing 50 μM of FAC, or 60 μM of DFP, CIK cells were harvested for the detection of mRNA expression. The results showed that the addition of FAC but not DFP significantly upregulated the expression of *CiCAT* and *CiSOD1* at the early stage, suggesting iron could aggravate oxidative stress in CIK cells, as seen in Figure 8C,D. Interestingly, we found that the addition of FAC inhibited the expression of *inducible nitric oxide synthase* (*iNOS*), as seen in Figure 8E, suggesting that iron enhanced intracellular oxidative stress through inducing the production of ROS rather than nitric oxide. Collectively, upon GCRV infection, *CiTfR1* intensifies intracellular oxidative stress by a mechanism involving in iron accumulation in CIK cells.

### 2.8. CiTfR1 but Not CiTf Overexpression Promotes CIK Cell Proliferation

**Figure 9:**
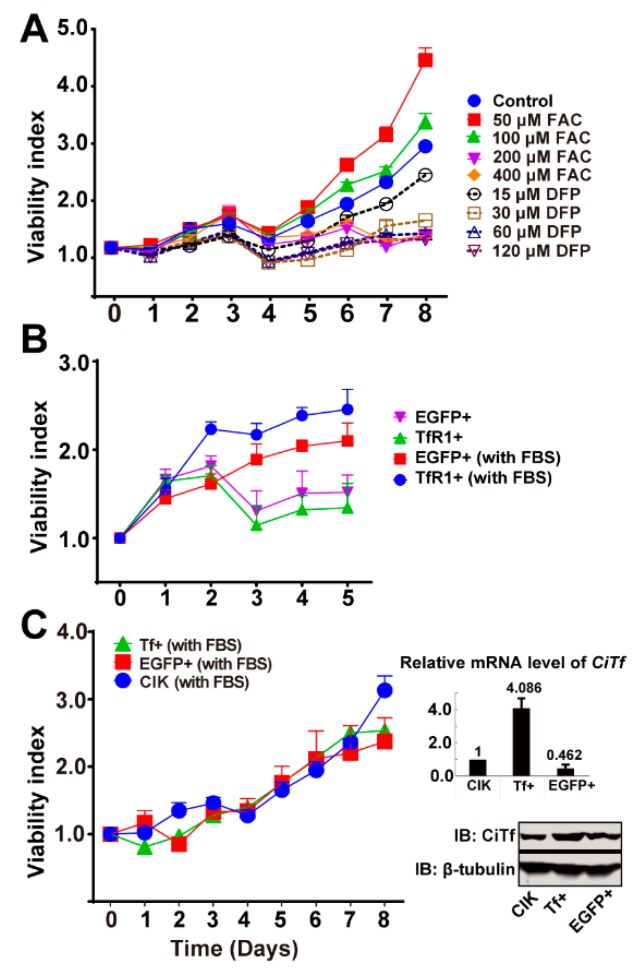
Both iron and *CiTfR1*, but not *CiTf*, facilitate the growth of CIK cells. (**A**) The growth curves of CIK cells cultured in medium with FBS and different concentrations of FAC, or DFP. (**B**) The growth curves of EGFP+ and TfR1+ cells cultured in medium with FBS or without FBS. (**C**) The growth curves of EGFP+ and Tf+ cells cultured in medium with FBS. All the data in the growth curves were obtained by MTT assay. Relative *CiTf* mRNA levels in the three sorts of cells were detected by RT-qPCR, in which *EF1α* severed as the reference gene. The protein levels were measured by WB. Data in the growth curve and overexpression verification represent mean ± SD of three independent experiments.

Iron can promote cell proliferation [6]. The present results showed that 50 or 100 μM of FAC in the medium promoted the proliferation of CIK cells, whereas 200 or 400 of μM FAC or any concentration of DFP in the medium were deleterious to the proliferation of CIK cells, as seen in Figure 9A. Since *Tf* and *TfR1* transfer iron into cells, we questioned whether *CiTf* and *CiTfR1* could promote the proliferation of CIK cells. The present results showed that TfR1+ cells grew better than EGFP+ cells with fetal bovine serum (FBS) in the medium, as seen in Figure 9B. Considering the insensitive expression of *CiTf* to GCRV infection, we deduced that *CiTf* overexpression could not promote the proliferation of CIK cells. To confirm this inference, we established a stable *CiTf* overexpression CIK cell line, called Tf+, as seen in Figure 9C. Subsequently, we verified that *CiTf* overexpression did not affect the proliferation of CIK cells, as seen in Figure 9C. Altogether, *CiTfR1* overexpression, but not *CiTf* overexpression, facilitates the proliferation of CIK cells.

## 3. Discussion

Given the crucial physiological function of iron in both hosts and microbes, the iron-withholding strategy of hosts is a potent immune response against various pathogen infection [5]. With the increasing research on iron-withholding strategies in plants and mammals, the relationship between iron metabolism and diseases of aquatic animals has drawn increasing attention. Studies have proved that pathogen infection, including fungal, bacterial, and viral, leads to extracellular and intracellular iron deficiency through iron-withholding mediated by ferritin and *Tf* [10,11,22,24]. Therefore, the present study aimed to identify the relationship between GCRV infection and iron metabolism in *C. idella*. Since iron is mobilized continuously and stored in the whole animal body, the iron content and the expression of IMRGs in a given tissue are inconstant [37]. Accordingly, we found that the iron content and gene expression were labile in the control groups. Therefore, all the observed changes are based on the comparison with the control group in the present study. Our results revealed that GCRV infection led to the iron accumulation in vivo and in vitro, as seen in Figure 1 and Figure 2, and that hepatopancreas as iron storage could accumulate iron at the early stage of GCRV infection and release iron into serum gradually, as seen in Figure 1. 

Considering iron overload is detrimental to host cell response against viral infection and beneficial to pathogen infection [6], we initially speculated that iron was indispensable to GCRV infection and that the iron increase in vivo and in vitro was actuated by GCRV. Therefore, we cultured CIK cells within different mediums containing different concentrations of FAC and then challenged with GCRV to verify the positive effect of iron on GCRV infection. Unexpectedly, addition of 50 μM FAC in cell culture medium reduced the load of GCRV and promoted cell survival, as seen in Figure 8, and upregulated the expressions of *CiCAT* or/and *CiSOD1* genes in CIK cells and macrophages, as seen in Figure 8 and Figure S4, implying that iron is detrimental to GCRV infection and that it can enhance the intracellular oxidative stress. Combining previous findings that HCV infection resulted in iron overload and that iron could inhibit HCV replication [38], we thought that the iron accumulation was actively regulated by host cells to fight against GCRV invasion. Interestingly, we found that an extra addition of 50 μM FAC in the medium downregulated the expression level of the GCRV *VP4* gene, as seen in Figure 8A, in CIK cells. With this, it is concluded that intracellular iron accumulation is a strategy of *C. idella* against GCRV infection, the aim of which is to stimulate oxidative stress or antiviral autophagy to impede viral infection [39]. This antiviral response is different from the well-known iron-withholding strategy, which may be because GCRV is a dsRNA virus whose replication does not require DNA synthesis that needs various iron-requiring proteins [40]. By this character of GCRV, the iron-withholding strategy may be inapplicable for grass carp to fight against GCRV infection, and alternatively, grass carp exchanges the iron-withholding strategy for the iron-accumulation strategy. As Figure 8A shows, an iron chelator did not decrease the virus load. Therefore, the antiviral response of iron accumulation may be an effective way for the host to fight against the invasive RNA virus. 

To clarify the mechanism by which GCRV infection induces iron accumulation, we detected the expression changes of major IMRGs upon GCRV infection in vivo and in vitro. The results showed that GCRV infection led to dysregulation of those genes, as seen in Figure 2 and Figure S1, suggesting that GCRV infection is associated with the iron metabolism in *C. idella*. More specifically, from the aspect of a single cell, two conceivable mechanisms may be responsible for the iron accumulation upon GCRV infection: One is the reinforcement of iron absorption, and the debility of iron efflux. According to the research results in mammals, iron absorption is mainly mediated by *TfR1*, while iron efflux is mainly mediated by *Fpn1* [13]. In fact, it is true in teleost [30,41]. We found that GCRV infection led to the increased expression of *CiTfR1* and reduced expression of CiFpn1, as seen in Figure 3, which might reduce iron accumulation. Surprisingly, *CiTf*, the ligand of *CiTfR1*, was not upregulated by GCRV infection in vivo and in vitro, as seen in Figure 3). Since fish *Tf* was early identified as one of the factors that mediate teleost macrophage activation and antimicrobial functions [42], this result may indicate that *Tf* does not directly participate in the antiviral immune response in grass carp. However, these results raise a question of whether, without *Tf* upregulation, only the upregulation of *CiTfR1* is sufficient for iron accumulation. We found that *CiTfR1* overexpression did not increase intracellular LIP in CIK cells without GCRV infection, while it increased intracellular LIP upon GCRV infection, and this increased effect could be inhibited by *CiTfR1* blockade or degradation, as seen in Figure 4. *CiTfR1* overexpression did not affect intracellular LIP in non-GCRV-infected cells, maybe due to the homeostasis of cells mediated by IMRGs. It is similar to the previous study that the sole overexpression of *TfR1* could not increase intracellular iron content [43]. After GCRV infection, the homeostasis was broken, thus the overexpressed *TfR1* induced intracellular accumulation. The present results are similar to the outcome of chronic HCV infection, wherein the upregulated *TfR1* is responsible for hepatic iron accumulation [44]. The observation that Ferristatin II or anti-CiTfR1 serum treatment impaired the *CiTfR1* overexpression-induced intracellular LIP increase indicated that *CiTfR1* played a positive role in the intracellular iron accumulation upon GCRV infection.

While *TfR1* is broadly documented as a transporter of iron, its other role as a virus entry factor has been identified [45]. Thus, we inferred that *CiTfR1* could facilitate the cell entry of GCRV, and the upregulated expression of *CiTfR1* was an outcome forced by GCRV, whilst intracellular iron accumulation was an incidental effect of *CiTfR1* expression. However, the results demonstrated that *CiTfR1* was unable to assist GCRV infection, as seen in Figure 5, but played a positive role in the antiviral immune response, as seen in Figure 6. Unexpectedly, we observed that the expression of *VP4* and *VP56* was high in negative-serum-treated CIK cells, but the survival ability of the negative-serum-treated cells was strong at the early stage of GCRV infection. Since cell survival was partially determined by the contest between cell proliferation and virus-induced cell death, the survival of cells may not necessarily reflex the decrease of the virus. We found that *CiTfR1* promoted cell survival, and in contrast, Ferristatin II or anti-CiTfR1 serum treatment showed an opposite effect on the cell survival upon GCRV infection. These results indicated that *CiTfR1* played a positive role in antiviral response. *TfR1* takes part in immune responses in several manners. For instance, *TfR1* contributes to the activation and proliferation of T cells [46,47]. However, that the question remains regarding by which mechanism *CiTfR1* regulates the antiviral immune response in teleost.

Herein, three probable mechanisms are proposed: (1) As discussed above, *CiTfR1* induces intracellular oxidative stress through iron accumulation; (2) because proliferating, metabolically active cells need iron to synthesize macromolecules [6], *CiTfR1* imports iron to promote CIK cell proliferation; (3) *CiTfR1* can provoke the IFN-I system. Along with these three hypotheses, we evidenced that *CiTfR1* overexpression not only enhanced intracellular oxidative stress in CIK cells, as seen in Figure 7, but also promoted the proliferation of CIK cells upon GCRV infection, as seen in Figure 9. Although a previous study found that iron overload, apoptosis, and oxidative stress were induced upon GCRV or infectious pancreatic necrosis virus infection in fish [48,49], the mechanisms for those findings are unclear. The present study reveals that the oxidative stress caused by GCRV infection depends in part on *CiTfR1* upregulation. More interestingly, conventional views hold that intracellular iron overload results in apoptosis, ferroptosis, and cell death [18,19], while the present study found that moderate addition of iron in CIK cells did not cause cell death, but promoted cell proliferation. It is worth mentioning that the overexpression of *CiTfR1* rather than *CiTf* promoted the growth of CIK cells, suggesting that *CiTfR1* is the dominant mediator for CiTf-TfR1-mediated iron import in CIK cells. For the third hypothesis, we chose *CiIFN1* and *CiIFN3* as representatives of IFN-I to estimate the relationship between the *CiTfR1* and IFN-I systems, because they are principal members with high expression in the IFN-I family in *C. idella* [50]. Unexpectedly, *CiTfR1* was unable to provoke the mRNA expression of either *CiIFN1* or *CiIFN3* at the early stage, implying that the positive role of *CiTfR1* in antiviral response is not directly associated with IFN-I.

**Figure 10:**
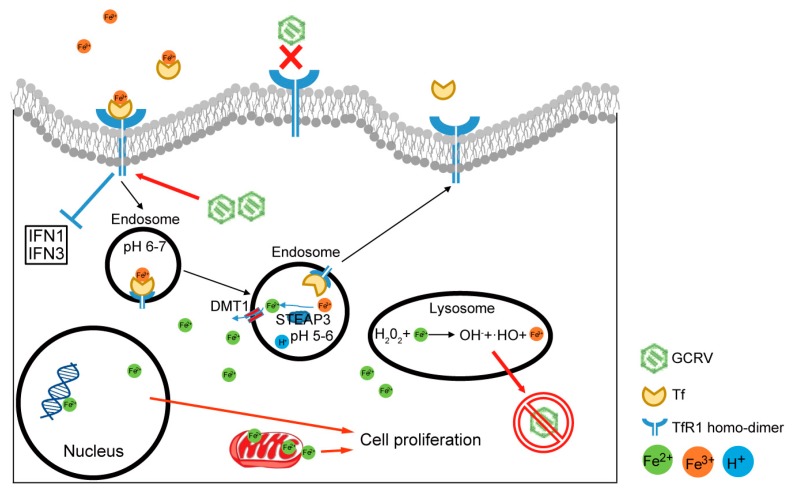
Schematic representation of *CiTfR1* inhibiting virus replication. Upon GCRV infection, *CiTfR1* is unable to facilitate GCRV cell entry (red X), but imports more iron into cells to enhance intracellular oxidative stress and promote cell proliferation, aiming at suppressing GCRV infection (red arrows and forbidden symbol). Meanwhile, *CiTfR1* has a weak inhibitory effect on the expression of *CiIFN1* and *CiIFN3* (blue block line).

Collectively, as illustrated in Figure 10, the present study demonstrates that *CiTfR1* is not a helper of GCRV infection, but it induces the accumulation of intracellular iron to promote cell proliferation and enhance intracellular oxidative stress, and eventually protects cells from virus infection. The present study uncovers a novel and IFN-I-independent antiviral immune response in teleost. Since soluble iron is scarce in natural water, the present study implies that the right amount of iron addition in feeds may be a possible method to increase the body and intracellular iron for grass carp fighting against GCRV infection.

## 4. Material and Methods

### 4.1. Fish, GCRV, and Challenge Experiment

Healthy *C. idella* of approximately 10 cm body length were obtained from the Freshwater Aquaculture Collaborative Innovation Center of Hubei Province, China. This study was carried out in strict accordance with the recommendations in the Guide for the Care and Use of Laboratory Animals of the National Institutes of Health and approved by the Animal Ethics Committee of Huazhong Agricultural University. A total of 180 fish were averagely distributed into the six tanks (30 fish in each tank), of which, three tanks were used for the challenge experiment, and another three tanks were served as the control group. Initially, fish were temporarily reared in those tanks filled with aerated freshwater at 28 °C for adaptation. During that time, fish were fed twice a day with the commercial feed. After seven days of acclimation, fish were employed for the following challenge experiment.

GCRV 097 strain (Type II GCRV, 3.63 × 10^7^ TCID_50_/mL) was propagated in CIK cells and stored at −80 °C. The GCRV challenge experiment was operated according to the related procedure in our previous study [51], and every effort was made to minimize the suffering of the fish. Briefly, by intraperitoneal and intramuscular injection, fish in the experimental group were challenged with GCRV at a dose of 100 μL per gram body weight, while fish in the control group were injected with sterile PBS at a dose of 100 μL per gram body weight. Considering that one of the symptoms of sick fish is anorexia, to simulate the natural condition of diseased fish and keep the water clean, no feedstuff was fed into those tanks, including control and experimental groups, until the end of this challenge experiment (seven days later). During the challenge experiment, water in those tanks were aerated continuously and changed every two days.

Grass carp with obvious symptoms of hemorrhagic disease, such as congestion or hemorrhage in muscle, operculum, fin base, intestine, and air bladder, were collected and sacrificed for sampling. Intestine samples were collected for RNA isolation. Blood samples were collected and divided into two parts: a fraction of samples was preserved in 1 mL of RNAiso Plus (Takara, Kusatsu, Japan) at ‒80 °C until RNA isolation, while the rest samples were directly preserved at 4 °C for the detection of serum iron content. Hepatopancreas samples were collected and divided into three parts: one was for the RNA isolation, one was preserved in Formalin solution for the histologic section, and the rest was for the detection of iron content. Simultaneously, samples were collected from the control group as well.

### 4.2. Histological Analysis and Prussian Blue Staining

Hepatopancreas samples were fixed for 18–24 h in 10% neutral-buffered formalin and transferred to 70% ethanol. Samples were then processed, embedded, sectioned at 4–6 μm. Paraffin sections of the same sample were classified into two groups: one group was stained with hematoxylin and eosin for pathological observation; another group was stained for ferric iron with Prussian Blue Stain (Perls’ iron) (Sigma-Aldrich, St. Louis, MO, USA), according to the protocol described before [22]. Stained sections were observed under a light microscope (Eclipse E100, Nikon, Japan) equipped with a digital camera (600D, Canon, Japan).

### 4.3. Cell Culture

CIK and L8824 cell lines were provided by the China Centre for Type Culture Collection. GCRV-resistant/susceptible phenotypic CIK cells were previously sorted and preserved in our laboratory [35]. Cells were cultured in DMEM (CIK cells) or M199 (L8824 cells) medium (Gibco, Carlsbad, CA, USA), supplemented with 10% FBS (Gibco), 100 U/mL penicillin (Sigma-Aldrich), and 100 U/mL streptomycin (Sigma-Aldrich), and maintained at 28 °C in a humidified atmosphere of 5% CO_2_ incubator (Thermo Scientific, Waltham, MA, USA). Macrophage isolation, culture, and Giemsa staining were conducted according to the detailed procedure described before [52].

### 4.4. Expression Vectors/Recombinant Plasmids

The open reading frames (ORFs) of *CiTfR1* and *CiTf* gene were obtained from the transcriptome database of *C. idella* and confirmed by aligning with the sequences in GenBank (Accession No. FJ613322 and AY383546, respectively) [53]. The appropriate full-length ORFs were amplified from the cDNA derived from hepatopancreas in *C. idella*, respectively. For prokaryotic expression, the signal peptide- and transmembrane domain-coding sequence-truncated ORF of *CiTfR1* was cloned into the *Xho*I/*Psy*I sites of the pGEX-4T-1 vector (Novagen, Darmstadt, Germany), while the full-length ORF of *CiTf* was cloned into the *Kpn*I/*Bam*HI sites of the pET-32a(+) vector (Novagen). For overexpression in CIK cells, the full-length ORFs of *CiTf* and *CiTfR1* were cloned into the *Kpn*I/*Apa*I and *Kpn*I/*Hin*dIII sites of the pCMV-eGFP-CMVs vector, respectively, as described in previous study [54]. The 5′-flanking sequence information of *CiTfR1* was obtained from the genome database of *C. idella* [55]. To verify the promoter activity, we constructed the pTfR1pro-EGFP plasmid by substituting CMV promoter with 5′-flanking sequence of *CiTfR1* (2439 bp) in the *Xho*I/*Hin*dIII sites of pCMV-eGFP, as described in previous study [54]. Subsequently, the promoter activity-validated 5′-flanking sequence was inserted into the *Bgl*II/*Hin*dIII sites of the pGL3-basic luciferase reporter vector (Promega), and the constructed plasmid was named as pTfR1pro-Luc. As a positive control, the CMV promoter was inserted into the pGL3-basic luciferase reporter vector as well, and we named the constructed plasmid pCMVpro-Luc. All the restriction endonucleases were obtained from Thermo Fisher Scientific. Primers used for constructs are listed in the Table S1. All the PCR amplicons were validated by Sanger sequencing. All the synthetic primers and DNA sequences were obtained from AuGCT Biotechnology Co., LTD (Wuhan, China).

### 4.5. Polyclonal Antisera Preparation and Commercial Abs

Considering that commercial anti-CiTfR1 and anti-CiTf Abs did not work well in *C. idella*, anti-CiTfR1 and anti-CiTf polyclonal Abs needed to be prepared. To address this, pGEX-4T-1-TfR1 and pET32a(+)-Tf plasmids were transformed into the *Escherichia coli* BL21 and BL21(DE3) cells (Novagen), respectively. Then, *CiTfR1* and *CiTf* proteins were expressed in vitro and purified. With six weeks of immunization on New Zealand rabbits, antiserum samples were collected. The titer and specificity of these antiserum were identified, according to the protocol mentioned previously [56]. Polyclonal anti-GCRV-VP56 Ab was previously prepared in our laboratory [57]. Anti-β-tubulin primary rabbit polyclonal Ab (ab6046) was obtained from Abcam, Cambridge, United Kingdom. IRDye^®^ 800CW Donkey anti-Rabbit-IgG and anti-Mouse-IgG (H+L) secondary Abs were purchased from LI-COR Bioscience, Lincoln, NE, USA.

### 4.6. Transfection, Infection, and Luciferase Activity Assay

To establish stable overexpression cells, we transferred 0.8 μg of either pTf, pTfR1, or pdCMV vector (vehicle control) into CIK cells by using FuGENE^®^ 6 transfection reagents (Promega, Madison, WI, USA), respectively, according to the instruction manual. Due to the low efficiency of transfection, we conducted the positive screening, as described previously [54], with some modification. More specifically, after we observed the green fluorescence in transfected cells by using a fluorescence microscope (Leica, Wetzlar, Germany), the cells were cultured in the medium containing 400 μg/mL of geneticin (G418, from Sigma-Aldrich). The medium was replaced every two days. Approximately two weeks later, positive cells formed some colonies, and each colony was picked into an independent well of a 24-well plate for separate culture. Then, the monoclonal cells overexpressed the target gene were further cultured for the subsequent experiments. 

For GCRV infection or poly(I:C) (Sigma-Aldrich) stimulation, cells were seeded into cell plates before infection. After washing the monolayer cells thrice with fresh serum-free DMEM, serum-free DMEM with 1.0 multiplicity of infection (MOI) of GCRV or 5 μg/mL of the final concentration of poly(I:C) was added into the wells of the experimental group, while serum-free DMEM with commensurate PBS was added into the wells of the control group. 

To identify the promoter activity of the *CiTfR1* promoter, pTfR1pro-EGFP was transfected into CIK cells, and then the expression of EGFP was assessed by imaging with a fluorescence microscope. For luciferase reporter assays, CIK cells were seeded in 24-well plates for overnight culture (5 × 10^5^/well), followed by cotransfection with the pTfR1pro-Luc and the pRL-TK vector (internal control reporter vector) at a ratio of 20:1 by using FuGENE^®^ 6 transfection reagents. Luciferase activities were measured by using the Dual-Luciferase Reporter Assay System (Promega) and a luminometer (GloMax 20/20, Promega). Data were normalized to the amounts of Renilla luciferase activities according to the protocol.

### 4.7. Cell Proliferation Assays

Cell proliferation was assessed by using 3-(4,5-dimethylthiazol-2-yl)-2,5-dimethyltetrazolium (MTT) method. MTT was obtained from Beyotime (Shanghai, China). Briefly, cells were seeded into 96-well plates for overnight culture (1× 10^4^/well). At the scheduled time, the OD value of each well was measured by a microplate reader (Infinite F200, Tecan, Männedorf, Switzerland) at the absorbance of 490 nm. Data were presented as viability index, which was calculated as the ratio between the OD value measured at the corresponding time-point and the OD value measured at the beginning of the experiment, namely, viability index = (OD value at indicated time-point)/(OD value at day 0).

### 4.8. Real-Time Quantitative PCR (RT-qPCR) Assay

For RT-qPCR assay, total cellular RNA was isolated by using RNAiso Plus (Takara) according to the manual. The quality of all the isolated RNA samples was measured by using a microplate spectrophotometer (BioTek, Winooski, VT, USA). The OD260/280 values of all the RNA samples were between 1.8 and 2.0. Subsequently, RNA samples were digested by RQ1 RNase-Free DNase (Promega). After that, 2 μg of each RNA sample was reverse-transcribed into cDNA by using M-MLV reverse transcriptase kit (Promega). All the procedures followed the instruction manuals. The SYBR Premix Ex Taq II kit (Takara) and a LightCycler 480 II Real-time PCR system (Roche, Basel, Switzerland) were used according to the instruction manuals. Primers are listed in Table 1. Relative target mRNA expression was calculated as the ratio of the real-time PCR signal of the particular target mRNA to that of the *EF1α* (for cells) or *18S rRNA* (for individuals) mRNA. Data were analyzed using the 2^−ΔΔCt^ or 2^−ΔCt^ (for the analysis of the *VP4* mRNA expression) method, as described before [58].

### 4.9. Western Blot (WB) Assay

At the scheduled time points, cells in six-well plates were washed with PBS and lysed in lysis buffer (Beyotime) supplemented with protease inhibitor cocktails (Sigma-Aldrich). After clarification by centrifugation at 12,000 rpm for 15 min, 30 μg of supernatant proteins were separated by 8% SDS-PAGE. The separated polypeptides were electroblotted onto nitrocellulose (NC) filter membranes (MilliporeSigma, Burlington, MA, USA) and further incubated with appropriate primary and secondary Abs. An Odyssey^®^ CLx Imaging System (LI-COR) was used for protein detection. For hybridization, the anti-CiTfR1 and anti-CiTf antisera were diluted at 1:1000, commercial primary Abs at 1:5000, secondary Abs at 1:10,000. 

### 4.10. Indirect Immunofluorescence Assay

Slides were fixed with 4% formaldehyde. Then the cells were incubated with primary polyclonal Ab against *CiTfR1* or negative serum (rabbit serum immunized with Freund′s adjuvant (Sigma-Aldrich)) at a 1:200 dilution overnight at 4 °C. Conjugated secondary Ab, Cy3-conjugated goat antirabbit Ab was incubated at a 1:300 dilution for 50 min at room temperature. Nuclei were stained with 4′,6-diamidino-2-phenylindole (DAPI) (Sigma-Aldrich), and images were taken with an UltraVIEW VoX 3D Live Cell Imaging System (PerkinElmer, Waltham, MA, USA).

### 4.11. Antiviral Activity Assay

Cells were seeded into 96-well or 48-well plates for overnight cultivation, and then the cells were infected with GCRV. Meanwhile, cells were treated with either 1 % DMSO (Sigma-Aldrich), 100 μM Ferristatin II (Sigma-Aldrich), 1% anti-CiTfR1 serum (preincubated at 56 °C for 30 min), or 1% negative serum (preincubated at 56 °C for 30 min). At 24 h p.i., cells were fixed with 4% paraformaldehyde for 10 min at room temperature. Cells in the 96-well plate were directly photographed by using an inverted fluorescence microscope (Ti-S, Nikon).

### 4.12. Oxidative Stress Measurement

Cells were seeded into 12-well plates for overnight culture, followed by appropriate treatments. At scheduled time points, cells were harvested for the measurement of oxidative stress markers, namely, total *CAT* activity and *CAT* mRNA expression, total *SOD* activity and *SOD1* mRNA expression, total antioxidant capacity, and total GSH content, by using corresponding testing kits (Sigma-Aldrich) or RT-qPCR assays.

For the assessment of superoxide anion content, the dihydroethidium (DHE) method was employed. DHE can be excited to emit blue fluorescence. Once DHE permeates the plasma membrane, it can be oxidized by intracellular superoxide anion into ethidium which combines with the nucleic acid to emit red fluorescence. Based on this principle, the stronger oxidative stress, the higher value of red fluorescence in cells. For analysis, cells were seeded into 24-well plates (5 × 10^5^/well), and 5 μM of the final concentration of DHE (Sigma-Aldrich) was added into each well for 2 h incubation at dark. Subsequently, cells were harvested and fixed with 4% paraformaldehyde for 10 min at room temperature, and then the fluorescence intensity in each well was measured in a Multiscan Spectrum microplate reader (SpectraMax i3x, Molecular Devices, San Jose, CA, USA) by loading 100 μL cell suspension into a lightproof 96-well plate. Fluorescence value was measured using two different filters with excitation wavelength of 370 nm and 520 nm, and emission at 420 nm and 610 nm, for blue and red fluorescence, respectively. Meanwhile, the cell concentration of each cell suspension sample was measured by a flow cytometer (Cell Lab Quanta SC, Beckman Coulter, Brea, CA, USA).

### 4.13. Tissue Iron Content, Serum TIBC, and LIP Measurement

Iron contents in hepatopancreas samples were measured by using ICP-OES technology. Briefly, after weighing, each fresh hepatopancreas sample was allotted into a separate microwave digestion tank filled with 8 mL of concentrated nitric acid. Subsequently, these tanks were embedded into a microwave digestion system (MARS6, CEM, Matthews, NC, USA), and digested with the following procedure: 150 °C, 15 MPa for 4 min, and then 180 °C, 25 MPa for 5 min. After digestion, each sample was diluted with ultrapure water to 50 mL, and then the iron content in each sample was measured by an ICP-OES (Optima 8000DV, PerkinElmer), according to the operating instruction. Serum iron content and TIBC which represents serum *Tf* concentration [59] were directly measured by an automatic biochemical analyzer (AU5400, Beckman Coulter).

To accurately measure intracellular LIP, a fluorescence probe and a Multiscan Spectrum microplate reader (SpectraMax i3x, Molecular Devices) were employed. The technical principle and procedure were specified in the previous report [8,60]. Specifically, the cells were rinsed once with PBS buffer and incubated with serum-free cell medium containing 150 nM calcein-acetomethoxy (CA-AM; Invitrogen, Carlsbad, CA, USA) at 28 °C for 20 min. CA-AM rapidly permeates the plasma membrane, and once inside the cell, it is hydrolyzed to release the acetomethoxyl group and the fluorescent metal chelator calcein (CA). CA’s fluorescence is then quenched immediately by intracellular metals, including iron. Excess CA-AM that did not penetrate the cells was removed by washing once with serum-free cell medium and then twice with PBS. The cells were then lysed with 1/3 × PBS and scraped with a cell scraper. After incubating on ice for 20 min, the cell lysates were centrifuged at 16,000× *g* for 10 min at 4 °C to remove cell debris, and the protein content of the supernatants was measured by the Bradford method. CA fluorescence intensity (F_0_) was measured in Multiscan Spectrum microplate reader (SpectraMax i3x, Molecular Devices) by loading 100 μL supernatant into a lighttight 96-well plate and using a filter combined with an excitation wavelength of 488 nm, an emission wavelength of 518 nm, and a cutoff wavelength of 495 nm. After this initial measurement, a strong iron-specific chelator, 2,2′-bipyridine (BIP; Sigma-Aldrich), was added to a final concentration of 100 μM to strip the iron from the chelated CA. The increased CA fluorescence intensity (F_1_) was then measured by using the same filter combination. The change in fluorescence intensity (ΔF = F_1_ − F_0_) thus represents the amount of iron that was bound to the CA in the cell, i.e., the cellular LIP. In the final calculation, the ΔF value of each sample was normalized against the lysate’s protein concentration to eliminate the effects caused by the number of cells.

### 4.14. Data Analysis

Statistical analysis and graphical presentations were carried out by using GraphPad Prism software (version 6.0, San Diego, CA, USA). ImageJ software (Version 1.8.0, NIH, Bethesda, MD, USA) was employed for the density scanning of pictures of Prussian blue staining and each WB band. Unpaired Student’s *t*-test was used in the data analysis, followed by a Benjamini–Hochberg false discovery rate test with desired value of 5%, and *p*-value < 0.05 was considered to be statistically significant.

## Figures and Tables

**Table 1:** Primers for real-time quantitative PCR (RT-qPCR) analysis.

Gene	Primer Name	Forward Primer (5′–3′)	Primer Name	Reverse Primer (5′–3′)
*EF1α*	EF125	CGCCAGTGTTGCCTTCGT	ER126	CGCTCAATCTTCCATCCCTT
*18s rRNA*	18F99	ATTTCCGACACGGAGAGG	18R100	CATGGGTTTAGGATACGCTC
*Tf*	TFF83	AGTTACTATGTCGTGGCGGTTG	TFR84	ATCCAGCGTTGCGGTTCA
*TfR1*	TrF85	GATGATGAAATGGAGGCTAACG	TrR86	GGCAATGACAAATCCGCAG
*TfR2*	TrF87	GAGGAGACTTTGGGAATGTTGG	TrR88	CAGAGGACTGGAGTAGACGGAGA
*Ferritin*	FeF89	TCCTGTGCTTCGTGCGTGT	FeR90	ACCTTCAGTCCGTCCTCGTG
*Hepcidin*	HeF91	TGAAACACCACAGCAGAACGA	HeR92	CAGCCTTTGTTACGACAGCAGTT
*Fpn1*	FpnF157	ACTCTTCGCTGGCGTCATTG	FpnR158	TGGATTTGGTGCGAGGATGA
*Steap3*	SteF159	GGTGTGTAAATCGCATCCCAT	SteR160	TAGTCGCTCCGCATTAGAAGG
*DMT1*	DmtF161	TTCTCATTGACGAACAGCCAG	DmtR162	CAAAGGAAAAGAGCCACGGAT
*CAT*	CatF163	GCCATCTCCAACGGCAACTT	CatR164	CCAGACCTTAGTCAAATCAAACGG
*SOD1*	SodF165	TCCGCACTTCAACCCTTACAG	SodR166	ACTTTCCTCATTGCCTCCCTT
*IL-6*	IL6F189	ACAGCAGAATGGGGGAGTTATC	IL6R190	CTCGCAGAGTCTTGACATCCTT
*TNF-α*	TnfF169	GCTGCTGTCTGCTTCACGC	TnfR170	AGCCTGGTCCTGGTTCACTCT
*IFN-γ*	WF79	CAGCGAACACCTGAAACTAACA	WR80	CCATCCCAAAGTCATCAAACAT
*iNOS*	WF81	CGAATACGCAATGGGAGAAC	WR82	GTGTCATAGCCTTTGGAGTCATAA
*IFN1*	IF590	AAGCAACGAGTCTTTGAGCCT	IR591a	GCGTCCTGGAAATGACACCT
*IFN3*	IF435	TACATTTATAGAGACTGCGGGTGG	IR357	TGGAGTGTCTGGTAAACAGCCTT
*VP4*	VF146	CGAAAACCTACCAGTGGATAATG	VR147	CCAGCTAATACGCCAACGAC

## Supplementary Materials

It can be found at https://www.mdpi.com/1422-0067/20/23/5857/s1. Table S1: Primers for plasmid construction. Figure S1: GCRV infection affect the mRNA expression of IMRGs in grass carp macrophages. Figure S2: Observation results for verifying the promoter activity of the 5′-flanking region of *CiTfR1* by fluorescent microscopy. Figure S3: Preparation of rabbit anti-CiTfR1 polyclonal anti-serum and verification of the recognition specificity. Figure S4: *CiTfR1* enhances the intracellular oxidative stress in grass carp macrophages upon GCRV infection.
