# Supplementary material for: Transferrin Receptor 1-Associated Iron Accumulation and Oxidative Stress Provides a Way for Grass Carp to Fight against Reovirus Infection
Source: Int J Mol Sci. 2019 Nov 22;20(23):5857. doi: 10.3390/ijms20235857 (PMC6929055; doi:10.3390/ijms20235857)
Supplement: Supplementary file 1 [file ijms-20-05857-s001.pdf]

Table S1. for plasmid construction.

Primer name	Primer sequence (5'–3')	Constructs
TFF53	**ATCG***GGTACC*GAACACCTTACCAGCAAATCAA	pET-32a(+)-Tf
TFR130	**CG***GGATCC*CAATCTACTATGCAGCTATCCAAA	
TrF97	**CCG***CTCGAG*AGCACAGACAAAACTGAAATCAG	pGEX-4T-1-TfR1
TrR94	**TGTA***GACTGGGTC*TTAAAGAGGTAAGCTGAATCTGC	
TFF53	**TCG***GGTACC*GAACACCTTACCAGCAAATCAA	pTf
TFR54	**ATCG***GGGCCC*TAAAACAAGACCTAATCACATCCAT	
TrF251	**CGG***GGTACC*TGTCTAGGGTGCTGCGGTT	pTfR1
TrR508	**CCC***AAGCTT*TTAAAGAGGTAAGCTGAATCTGC	
TrF543	**CCG***CTCGAG*GACATTTGTTACCCCCTTGC	pTfR1pro-EGFP
TrR544	**TAC***AAGCTT*ACAACCGCAGCACCCTAG	
TrF651	**GA***AGATCT*AAATCTAAAGTTATCATACCTGGG	pTfR1pro-Luc
TrR652	**CCC***AAGCTT*GTCATAAATCTTACCAATGCTCTC	

Note: Italic letters indicate the accessional restriction enzyme cutting sites, bold letters indicate the protective bases.

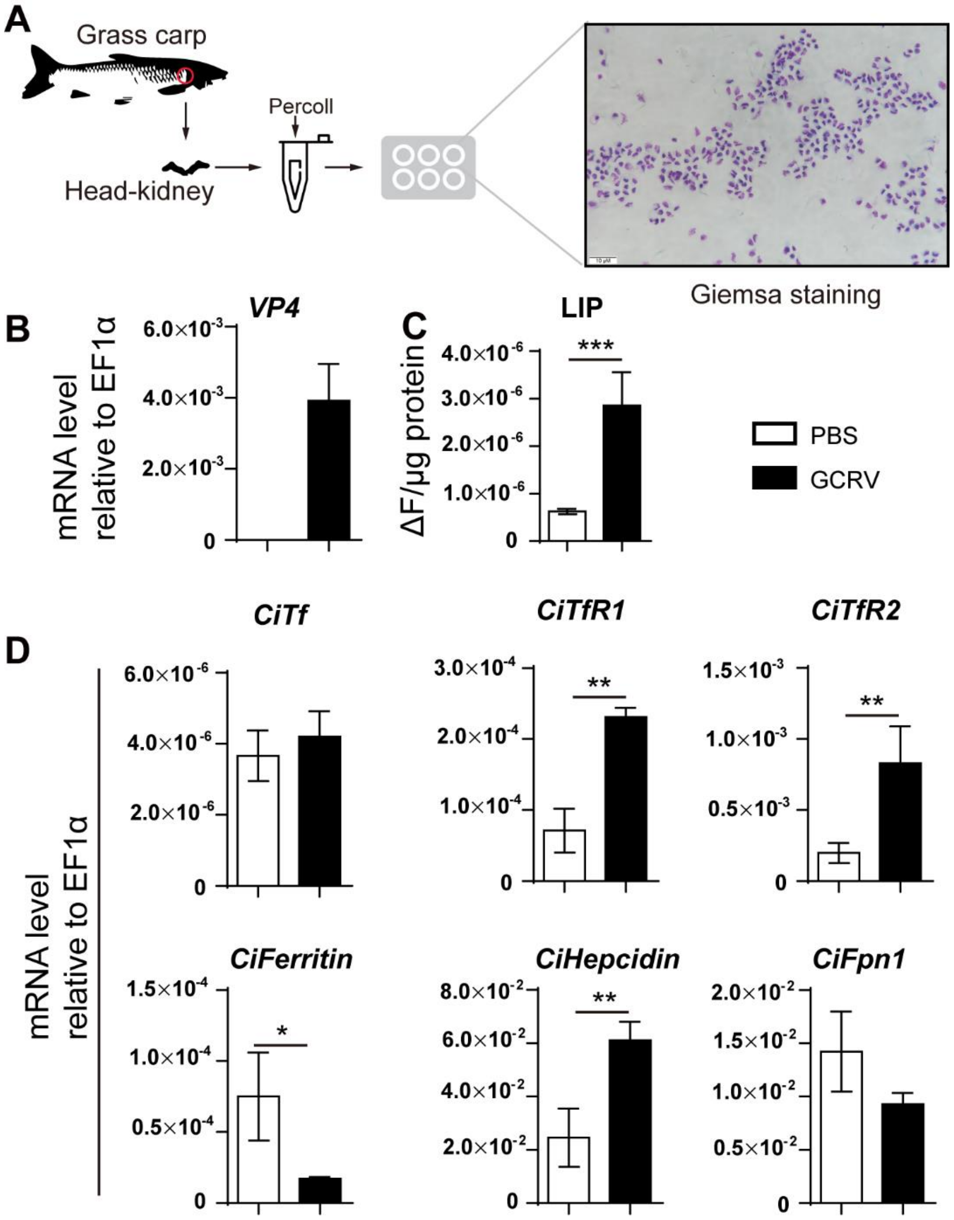

Figure S1. GCRV infection affect the mRNA expression of IMRGs in grass carp macrophages. (**A**) Diagram of macrophage isolation and the Giemsa staining (Scale bar = 10 μm). (**B-D**) 5×10^5 isolated macrophages were seeded into 12-well plates, and then infected with GCRV (MOI=1). Twelve hours later, cells were harvested for RNA isolation and subsequent RT-qPCR assay (B and D), or LIP measurement (C). Data of B and D are presented in relative expression units where *EF1α* was used to normalize all sample. Data represent mean ± SD of three independent experiments. *$p < 0.05$, **$p < 0.01$, *** $p < 0.001$.

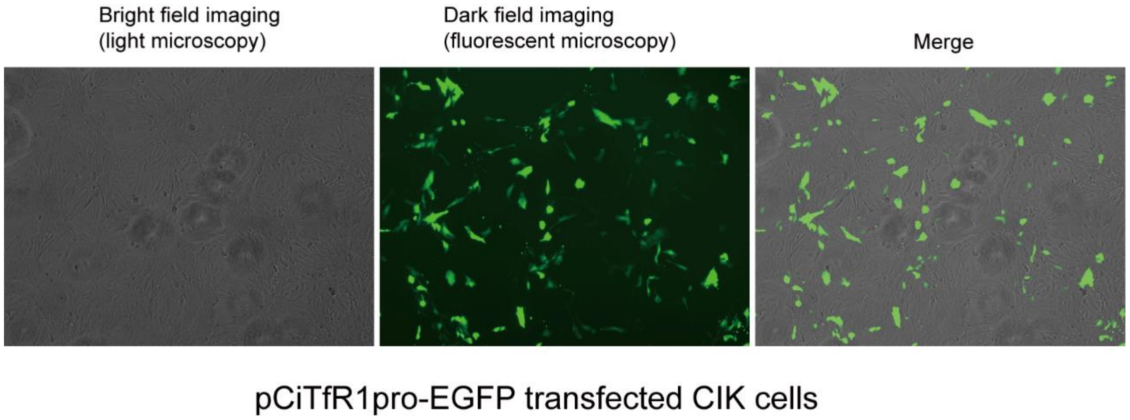

Figure S2. Observation results for verifying the promoter activity of the 5'-flanking region of *CiTfR1* by fluorescent microscopy. The CIK cells transfected with pTfR1pro-EGFP, were observed under a fluorescent microscopy. These green fluorophores are the EGFPs. Magnification: 10 × 10.

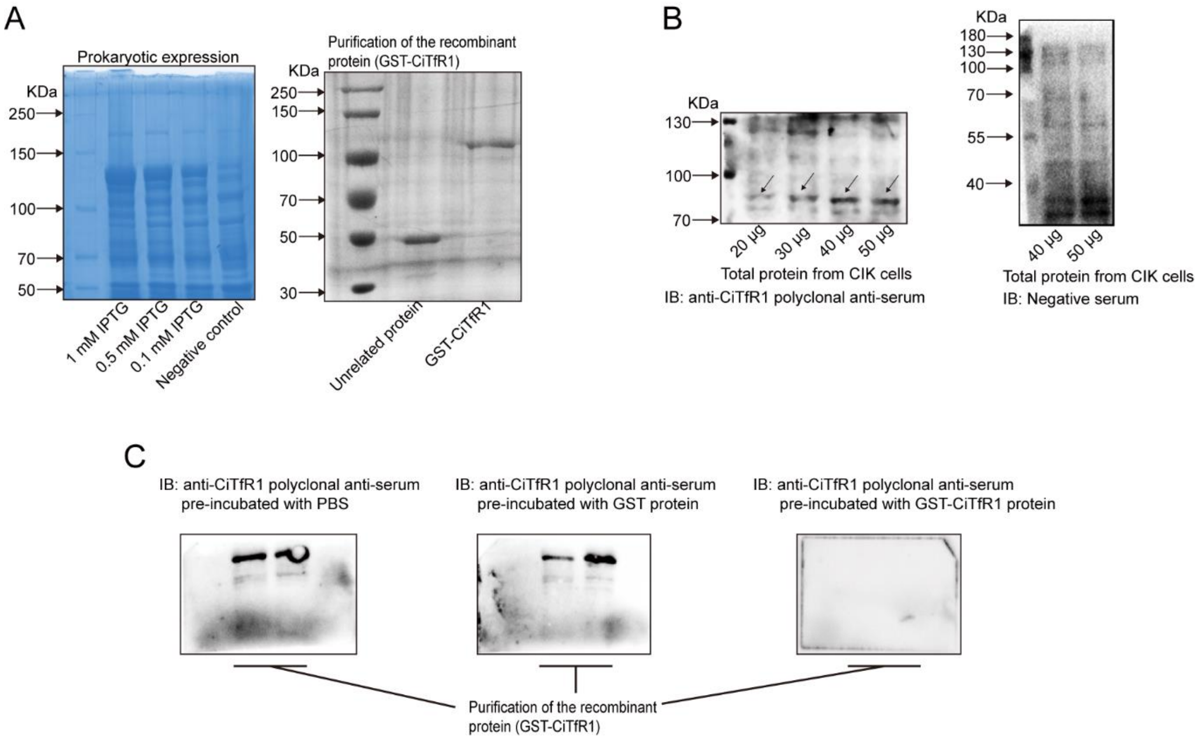

Figure S3. Preparation of rabbit anti-CiTfR1 polyclonal anti-serum and verification of the recognition specificity. (**A**) The truncated sequence of *CiTfR1* was expressed as GST-tagged fusion protein (GST-CiTfR1) in BL21 *E. coli* strain. The lysates of non-induced bacteria, i.e., Negative control, 0.1 mM, 0.5 mM, or 1 mM IPTG induced bacteria, and the purified protein were separated in 10 % SDS-PAGE gels and stained with Coomassie brilliant blue R-250. (**B**) CIK cells were seeded into a 6-well plate, and lysed by RIPA lysis buffer (1% Triton X-100, 1% deoxycholate, and 0.1% SDS) 24 h latter. The cytolysates were separated in 10 % SDS-PAGE gels, and then trans-blotted onto a NC membrane for WB analysis. (**C**) Polyclonal anti-CiTfR1 serum was separately incubated with either PBS, purified GST protein, or purified GST-CiTfR1 protein. Then, these pre-incubated sera were served as primary Abs to blot purified GST-CiTfR1.

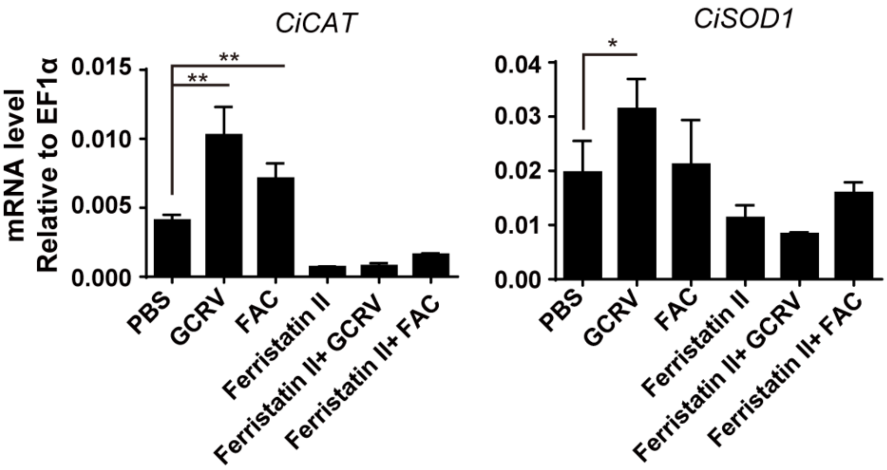

Figure S4. *CiTfR1* enhances the intracellular oxidative stress in grass carp macrophages upon GCRV infection. 5×10^5 isolated macrophages were seeded into 12-well plates, and then treated with the indicated treatments (concentrations of Ferristatin II and sera were 100 μM and 1%, respectively; MOI of GCRV is 1). Twelve hours later, macrophages were harvested for RNA isolation. The relative mRNA levels of *CiCAT* and *CiSOD1* were measured by RT-qPCR assay. Data are presented in relative expression units where *EF1α* was used to normalize all sample. Data represent mean ± SD of three independent experiments. *$p < 0.05$, **$p < 0.01$.
